# Metabolically conditioned media derived from bone marrow stromal cells or human skin fibroblasts act as effective chemoattractants for mesenchymal stem cells

**DOI:** 10.1186/s13287-017-0664-5

**Published:** 2017-09-29

**Authors:** Anastasia Gabrielyan, Elena Neumann, Michael Gelinsky, Angela Rösen-Wolff

**Affiliations:** 1 Department of Pediatrics, University Hospital Carl Gustav Carus, Technische Universität Dresden, Fetscherstraße 74, 01307 Dresden, Germany; 2 Centre for Translational Bone, Joint and Soft Tissue Research, Technische Universität Dresden, Fetscherstraße 74, 01307 Dresden, Germany; 3 Department of Internal Medicine and Rheumatology, Justus-Liebig-University Gießen and Kerckhoff-Klinik Bad Nauheim, Benekestraße 2-8, 61231 Bad Nauheim, Germany

**Keywords:** Mesenchymal stromal cells, Fibroblasts, Hypoxia, Migration, Tissue repair

## Abstract

**Background:**

The main goal of bone tissue engineering has been the generation of healthy bone in order to replace affected tissue. Therefore, optimized biomaterials are needed which allow the survival and growth of mesenchymal stem cells. Until now the key challenge in the clinical application of cell-based tissue engineering bone implants was poor diffusion of oxygen into the tissue, making functional blood vessel networks a necessity. With their ability to evolve into different cell types, to expand extensively in vitro, and to release paracrine soluble factors, bone marrow stromal cells (BMSC) are highly attractive for tissue engineering. During the last years hypoxia became a proven method to control proliferation, differentiation, and pluripotency of BMSC. Here we applied different methods to characterize metabolically conditioned media (MCM) in comparison to hypoxia conditioned media (HCM) and evaluated their ability to attract BMSC in 2-D migration assays.

**Methods:**

BMSC and fibroblasts of human origin were isolated and cultivated to obtain HCM and MCM. Both media were characterized by angiogenesis arrays, cytokine arrays, and ELISA for selected factors. 2-D migration tests were performed with Corning Transwell®-96 permeable support chambers with porous polyester membranes with a pore size of 8.0 μm.

**Results:**

Characterization of HCM and MCM revealed that the concentration of angiogenic factors was higher in MCM than in HCM. However, the chemoattractive capacity of MCM for BMSC was equivalent to that of HCM. HCM and MCM produced by human skin fibroblasts attracted human BMSC as efficiently as HCM and MCM produced by human BMSC.

**Conclusions:**

HCM and MCM have a high chemoattractive capacity for BMSC. Both conditioned media harbor high concentrations of angiogenic factors which are important for angiogenesis and cell migration. Both chemoattracting conditioned media can also be derived from skin fibroblasts which can easily be obtained from patients in individualized therapy approaches.

## Background

The potential for self-regeneration of bone tissue is not sufficient to regain the original function in the case of extensive lesions, osteoporosis, injury, or tumor resection. Hence, the main goal of bone tissue engineering has been the generation of healthy bone to replace affected bone tissue. Therefore, optimized biomaterials are needed which allow survival and growth of mesenchymal stem cells, a subset of bone marrow stromal cells (BMSC), with the ability to differentiate into osteoblasts and osteocytes. Another critical point is to attract endothelial cells in order to provide neovascularization.

With their ability to differentiate into different cell types, to expand extensively in vitro, and to release paracrine soluble factors, BMSC are highly attractive for tissue engineering [1, 2]. Human BMSC were shown to chemotactically respond to several factors, including platelet-derived growth factor (PDGF), vascular endothelial growth factor (VEGF), insulin-like growth factor (IGF-1), interleukin-8 (IL-8), bone-morphogenetic protein (BMP)-4, and BMP-7 [3]. They were observed to migrate toward the sites of injury in response to chemotactic signals in vivo [4]. BMSC are also able to secrete significant levels of chemoattractive agents like VEGF, monocyte chemoattractant protein-1 (MCP-1), macrophage inflammatory protein-1α (MIP-1α), MIP-1β, and monokine induced by IFN-γ (MIG) [5].

It is widely known that angiogenesis plays an important role in bone repair and wound healing. Wound healing is a complex process requiring cell migration, inflammation, angiogenesis, granulation tissue formation, reepithelialization, and extracellular matrix (ECM) remodeling. New blood vessel formation on the site of injury allows for cell migration and transport of nutrients to the site of injury. Studies have shown that promotion of vessel formation in an injury can influence bone healing as well as that some factors can cause delayed fracture healing [6]. MSC play an active role in this process and therapeutic application of MSC has been shown to enhance angiogenesis and improve wound-healing outcomes. For example, treatment of rat wounds with human umbilical cord MSC increased the levels of VEGF (one of the most potent proangiogenic factors), the density of microvessels, and cutaneous wound microcirculation [7]. Data from a study of rat adipose-derived stem cells implanted into rat wounds suggested that MSC could participate in vasculogenesis of wound healing through direct differentiation into vascular endothelial cells [8]. These same MSC also secreted significantly higher levels of the proangiogenic cytokines VEGF and hepatocyte growth factor [8]. In another study, Schlosser et al. [9] demonstrated that injected murine bone marrow MSC reduced arteriolar vascular resistance and increased functional capillary density in the vasculature of murine skin recovering from ischemia. The key finding in this study was the expression of proangiogenic factors such as VEGF by the MSC. Vertelov et al. [10] were able to show a high migration capacity of human MSC toward different angiogenic factors and cytokines such as HGF, PDGF, EGF, VEGF, bFGF, IGF-1, MIP-3β, MIP-1α, RANTES, SDF-1α, IL-1β, IL-6, IL-8, and TNF-α. Hence, optimized biomaterial will not only have to facilitate the migration and differentiation of MSC to the site of injury but also allow for angiogenesis at the site of implantation.

During the last years hypoxia became a proven method to control proliferation, differentiation, and pluripotency of BMSC [11–13]. Knowing that the secretion of VEGF from these cells can be upregulated by hypoxic conditions in a hypoxia-inducible factor-1 (HIF-1)-dependent way [14], we showed in our previous research that hypoxia conditioned media have a species-specific chemoattractive capacity for human, ovine, equine, and canine BMSC [15]. HCM contain attractors even more potent than VEGF alone and can therefore be used in many animal species without the need for purified proteins [15]. In our recent studies we were able to show for the first time a very potent alternative to HCM, which we termed “metabolically conditioned media” (MCM). Its generation is even easier and cheaper without the need for expensive hypoxia chambers. In migration assays we were able to prove its effective chemoattractive capacity for BMSC. Our second milestone was to confirm that HCM and MCM produced by human skin fibroblasts were also extremely potent chemoattractive agents, which takes us one step closer to individualized medicine as it is more feasible to obtain human skin fibroblasts than BMSC from patients in individualized therapy approaches. Here we present the characterization of HCM and MCM of different human cell sources and their composition of immunomodulatory cytokines and angiogenic factors, as those are crucial for cell migration and new vessel formation.

## Methods

### Isolation and cultivation of human BMSC

Human BMSC were provided by Translational Biomedical Research Group, Center for Regenerative Therapies, Dresden, Germany. To enrich human bone marrow stromal cells (BMSC) we performed density gradient centrifugation by Ficoll (Biochrom, Berlin, Germany). After centrifugation we used CD105 and CD271 MicroBead kits (Miltenyi Biotec, Bergisch Gladbach, Germany) for magnetic labeling and positive selection of the BMSC according to the manufacturer’s instructions to separate BMSC from possible residues. Thereafter, cells were cultured in T-175 flasks (Greiner Bio-One, Frickenhausen, Germany) in alpha medium (Biochrom) containing 10% fetal calf serum (FCS) (Sigma), 1% l-glutamine (PAA, Pasching, Austria), and 1% penicillin/streptomycin (PAA) in a humidified atmosphere with 20% O_2_, 5% CO_2_ at 37 °C (Thermo Scientific BBD 6220 CO_2_ Incubator; Omnilab, Bremen, Germany). After 4–6 days the culture medium was exchanged and thereafter every 3–4 days until the culture reached 80–90% confluence.

### Cultivation of human fibroblasts

Human male foreskin fibroblasts in passage 5 were kindly provided by the Department of Pediatrics, University Clinic Carl Gustav Carus, Dresden, Germany. Human osteoarthritis synovial fibroblasts in passage 3, which were isolated from patients with osteoarthritis of the knee, were provided by the Department of Internal Medicine and Rheumatology, University of Giessen. They were cultured in T-75 flasks (Greiner Bio-One) in Dulbecco’s Modified Eagle’s Medium—low glucose (Sigma-Aldrich, Munich, Germany) containing 10% FCS (Sigma), 1% l-glutamine (PAA), and 1% penicillin/streptomycin (PAA) in a humidified atmosphere with 20% O_2_, 5% CO_2_ at 37 °C (Thermo Scientific BBD 6220 CO_2_ Incubator; Omnilab).

### Generation of hypoxia conditioned media

Human BMSC were cultured in T-175 flasks in alpha medium containing 10% FCS, 1% penicillin/streptomycin (PAA), and 1% l-glutamine in a normoxic chamber until the cultures reached 80–90% confluence. Thereafter, media were exchanged and the cells were cultured for 24 h in alpha medium containing 5% FCS, 1% l-glutamine, and 1% penicillin/streptomycin. The flask was then transferred into a hypoxic chamber and cultured in a humidified atmosphere with 1% O_2_, 5% CO_2_ at 37 °C for 48 h (Thermo Scientific HERAcell 150i, Waltham, MA, USA). Thereafter, the supernatants (HCM) were collected, aliquoted into 1.5-ml tubes (Eppendorf), and stored at –80 °C to be used in migration assays. Control media were generated under normoxic conditions.

Human skin and osteoarthritis fibroblasts were cultured in T-75 flasks in low-glucose DMEM until they reached 90% confluence, and then the media were exchanged to alpha medium containing 5% FCS, 1% l-glutamine, and 1% penicillin/streptomycin. Generation of HCM was as already described.

### Generation of metabolically conditioned media

Human BMSC and human skin fibroblasts were cultured in T-175 flasks in alpha medium containing 10% FCS, 1% l-glutamine, and 1% penicillin/streptomycin (PAA) in a normoxic chamber until the cultures reached 80–90% confluence. Thereafter, the media were exchanged to alpha medium containing 5% FCS, 1% l-glutamine, and 1% penicillin/streptomycin and cells were cultured for 14 days without exchange of the medium. The supernatants (MCM) were collected, aliquoted into 1.5-ml tubes (Eppendorf), and stored at –80 °C to be used in migration assays.

### Human angiogenin, KGF, Pentraxin-3, Thrombospondin-1, TIMP-1, and uPA ELISA of HCM and MCM

In order to quantify the content of the angiogenic factors in human HCM and MCM, enzyme-linked immunosorbent assays (ELISAs) were performed according to the manufacturer’s instruction. ELISA kits for all angiogenesis factors were ordered from R&D (Abingdon, UK).

### Human angiogenesis array

The Human Angiogenesis Array kit (R&D Systems, Germany) is a rapid and sensitive tool to simultaneously detect the relative levels of expression of 55 angiogenesis-related proteins. Capture antibodies have been spotted in duplicate on nitrocellulose membrane. Human BMSC-derived HCM or MCM (B-HCM or B-MCM), skin fibroblast-derived HCM or MCM (F-HCM or F-MCM), and control media were mixed, 1 ml respectively, with a cocktail of biotinylated detection antibodies. As control media we used the supernatant, which was obtained from normally (no hypoxia, no starving) treated BMSC or fibroblasts. The sample/antibody mixture was then incubated with the Human Angiogenesis Array. Any protein/detection antibody complex present is bound by its cognate immobilized capture antibody on the membrane. Wash steps removed unbound material, and afterwards Streptavidin-HRP and chemiluminescent detection reagents were added. Light was produced at each spot in proportion to the amount of analyte bound. The positive signals on developed film were identified by placing the transparency overlay on the array image and aligning it with the reference spots in three corners of each array. Pixel density was analyzed with ImageJ (Wayne Rasband, NIH). The results were described in a table using “yes” and “–” (for no) in order to show the presence of each tested factor.

### Human cytokine array

The Human Cytokine Array (R&D Systems, Germany) is a rapid and sensitive tool to simultaneously detect relative expression levels of 36 human cytokines, which are spotted in duplicate on nitrocellulose membrane. B-HCM or B-MCM, F-HCM or F-MCM, and control media, 1 ml respectively, were mixed with a cocktail of biotinylated detection antibodies. The mixture was then incubated with the Human Cytokine Array Panel A membrane. A wash step was needed to remove unbound material, and then Streptavidin-HRP and chemiluminescent detection reagents were added. Light was produced at each spot in proportion to the amount of analyte bound. The positive signals on developed films were identified by placing the transparency overlay on the array image and aligning it with the reference spots in three corners of each array. Pixel density was analyzed with ImageJ (Wayne Rasband, NIH).

The results were described in a table using “yes” and “–” (for no) in order to show the presence of each tested factor.

### 2-D migration assay

The 2-D cell migration assays were performed in Corning Transwell®-96 permeable support chambers with porous polyester membranes with a pore size of 8.0 μm (Corning Incorporated Life Sciences, Munich, Germany). Human BMSC were resuspended at 5 × 10^4^ cells/ml in Alpha Medium supplemented with 1% l-glutamine, 1% penicillin/streptomycin, without BSA, and seeded into the upper chamber. HCM or MCM from corresponding BMSC and HCM or MCM generated from fibroblasts were used as the chemoattractive agent in the lower compartment. The 96-well plate was incubated overnight at 37 °C in a normoxic incubator. The cells on the upper membrane compartment were removed manually. The migrated cells adhering to the bottom side of the membrane and from the lower chamber were then dislodged by incubating the inserts in 0.25% trypsin for 4 min followed by treatment with trypsin neutralization solution (Promocell, Heidelberg, Germany). AlamarBlue® (Invitrogen, Darmstadt, Germany) was used to stain the cells overnight. Cell number was determined by fluorescence measurement at excitation 560 nm/emission 590 nm using a Tecan microplate reader (Männedorf, Switzerland). Results are described as the mean percentage of migrated cells over control cells; the latter show basal migration without chemotactic signal. Experiments for human B-HCM or B-MCM were repeated eight times, and those for F-HCM or F-MCM were performed four times.

### Ethics statement

All procedures were approved by the Ethical Commission EK261092009 of the Medical Faculty of Technical University Dresden.

### Statistical analysis

The *t*-test was performed using the program available online (http://www.daten-consult.de/forms/ttestunabh.html).

## Results

### Characteristics of HCM and MCM

In order to determine which angiogenic factors were released from BMSC and foreskin fibroblasts under hypoxic (hypoxia conditioned media, B-HCM) and starving (metabolically conditioned media, B-MCM) conditions we performed a Human Angiogenesis Array analysis. As shown in Table 1, different angiogenic factors were detected in human B-HCM and B-MCM. For further exact quantification by ELISA we decided to only select factors that were elevated, present in both conditioned media and control, and also factors which might be considered important for migration of cells. ELISA results of selected factors showed that all concentrations of these selected proteins were higher in both supernatants than in control media as well as each protein being more concentrated in MCM than in HCM (Table 2). Normoxic media (alpha medium with 5% FCS, 1% penicillin/streptomycin, and 1% l-glutamine) which were obtained from untreated BMSC (B-control, no hypoxia, no starving) served as control. Since we wanted to compare the migration potential of B-conditioned media and F-conditioned media, we additionally performed angiogenesis arrays for F-HCM and F-MCM. The analysis clearly showed that there are more angiogenic factors present in F-HCM and F-MCM than in control media (Table 1, right columns). Also Activin A, DPPIV, GM-CSF, HB-EGF, and HGF could only be detected in F-MCM. Angiopoietin-2, Artemin, Coaglutin Factor III, EG-VEGF, FGF basic, and Leptin were elevated in both F-conditioned media compared to respective control, while ADAMATS-1, Endoglin, FGF-7, TIMP-4, uPA, and VEGF-C were downregulated in F-HCM and could only be detected in F-MCM and F-control.

**Table 1:**
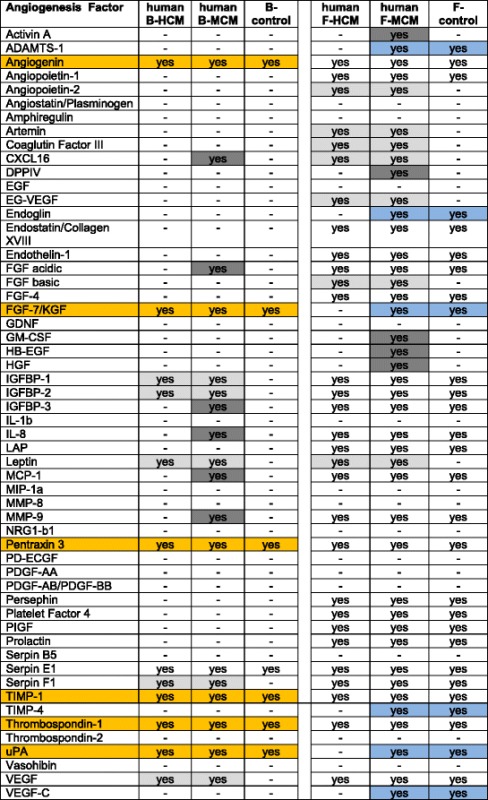
Results of human angiogenesis array for HCM, MCM, and control media obtained from BMSC and human skin fibroblasts

*White* no differences between control, HCM, and MCM, *light gray* elevated in HCM and MCM compared to control, *dark gray* elevated in MCM compared to HCM and control, *blue* elevated in MCM and control compared to HCM

*Orange* selected for further quantification (Table 2). The experiments were repeated twice with two different donors (total of four replicates, two replicated per one nitrocellulose membrane)

*B-control* control media derived from BMSC, *B-HCM* hypoxia conditioned media from BMSC, *B-MCM* metabolically conditioned media from BMSC, *BMSC* bone marrow stromal cells, *F-control* control media derived from skin fibroblasts, *F-HCM* hypoxia conditioned media from skin fibroblasts, *F-MCM* metabolically conditioned media from skin fibroblasts, *HCM* hypoxia conditioned media, *MCM* metabolically conditioned media

**Table 2:** Quantification of human angiogenesis factors in human HCM and MCM derived from BMSC

Media	Angiogenin (pg/ml)	KGF (pg/ml)	Pentraxin-3 (pg/ml)	Thrombospondin-1 (pg/ml)	TIMP-1 (pg/ml)	uPA (pg/ml)
B-HCM	625	452.3	68.2	673.7	12,500	4357.2
B-MCM	1250	10,002.9	92.6	929.4	14,800	5339.6
B-control	192	158.4	55.7	525.6	9700	90.14

*B-control* control media derived from BMSC, *B-HCM* hypoxia conditioned media from BMSC, *B-MCM* metabolically conditioned media from BMSC, *BMSC* bone marrow stromal cells, *HCM* hypoxia conditioned media, *MCM* metabolically conditioned media, *KGF* Keratinocyte Growth Factor, *TIMP-1* tissue inhibitor of metalloproteinases, *uPA* urinary Plasminogen Activator

Characterization of B-HCM and B-MCM also included semiquantitative analyses of cytokine factors in comparison to control media, as many of them might be harmful and reverse the effect of angiogenesis factors. B-control contained CCL2/MCP-1, CXCL12/SDF-1, IL-6, IL-8, MIF, and Serpin E1/PAI-1. In contrast, MIF was not present in B-HCM and IL-8 was not present in B-MCM (Table 3, left columns). Hence, under hypoxic and metabolic conditioning, the release of cytokines from BMSC was not upregulated but rather reduced while release of angiogenic factors was clearly upregulated (Table 2).

**Table 3:**
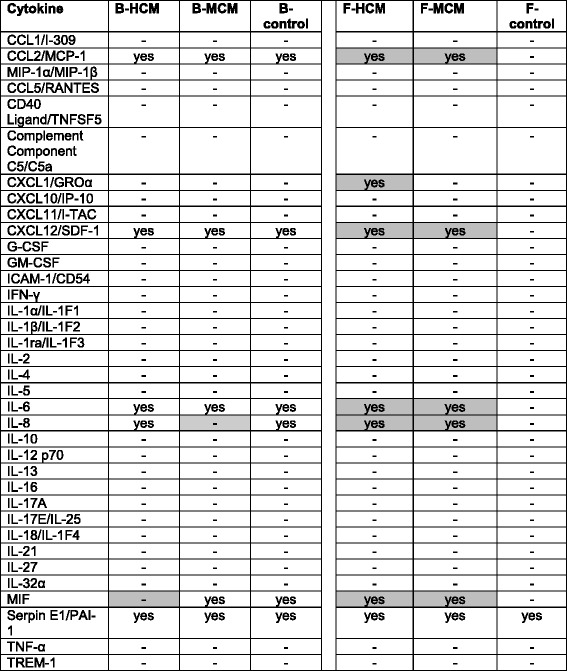
Results of human cytokine array analysis of HCM, MCM, and control media

The experiments were repeated twice with two different donors (total of four replicates, two replicated per one nitrocellulose membrane)

*B-control* control media derived from BMSC, *B-HCM* hypoxia conditioned media from BMSC, *B-MCM* metabolically conditioned media from BMSC, *BMSC* bone marrow stromal cells, *F-control* control media derived from skin fibroblasts, *F-HCM* hypoxia conditioned media from skin fibroblasts, *F-MCM* metabolically conditioned media from skin fibroblasts, *HCM* hypoxia conditioned media, *MCM* metabolically conditioned media

BMSC can serve as a potent source for chemoattractive conditioned media. However, for individualized therapy approaches we sought to find a method to obtain chemoattractive conditioned media from cells which do not need to be obtained by bone marrow aspiration. Hence, we focused our interest on human foreskin fibroblasts which can easily be obtained by skin biopsies of individual patients. Thus, HCM and MCM produced from skin fibroblasts (F-HCM, F-MCM, F-control) were also analyzed for the presence of cytokines (Table 3, right columns).

F-control contained only Serpin E1/PAI-1; however, F-HCM and F-MCM also contained CCL2/MCP-1, CXCL12/SDF-1, IL-6, IL-8, and MIF. CXCL1/GROα was only present in F-HCM and could not be found in any of the other media. Hence, under hypoxic and metabolic conditioning, skin fibroblasts’ release of cytokines and of angiogenic factor was clearly upregulated.

### 2-D migration of human BMSC

**Fig. 1:**
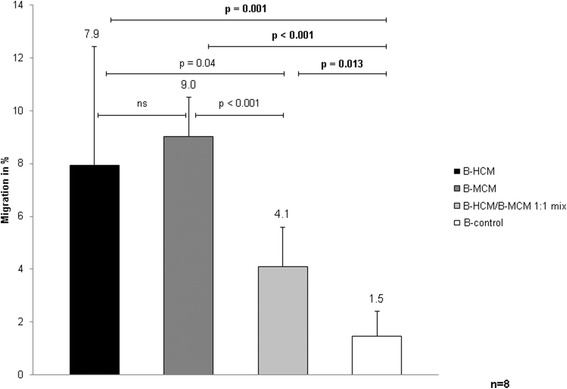
Migration (%) of human BMSC toward B-HCM, B-MCM, and a 1:1 mixture of both. Differences between migration of BMSC attracted by control (untreated) media, B-MCM, or B-HCM were evaluated using *t*-test. *p* < 0.05 statistically significant, ns not significant. Experiments were performed with different donors and different passages eight times (each time three replicates per sample). B-control control media derived from BMSC, B-HCM hypoxia conditioned media from BMSC, B-MCM metabolically conditioned media from BMSC, BMSC bone marrow stromal cells

In order to determine the capacity of B-HCM and B-MCM to attract BMSC of human origin, we performed migration assays. As shown in Fig. 1, in comparison to control media (B-control, untreated BMSC supernatant), human B-HCM were significantly more potent to attract BMSC (*p* = 0.001). Migration was increased 5.3-fold. Human B-MCM increased the cell migration 6-fold (*p* < 0.001), showing that both conditioning regimes were comparable without any significant difference. A 1:1 mixture of human HCM and MCM seemed a promising approach since we found that each of them harbor different angiogenesis factors (Table 1). However, the combination of both was considerably less effective. These results indicate that MCM is a promising chemoattractive substance which is cheaper and easier to produce than HCM.

**Fig. 2:**
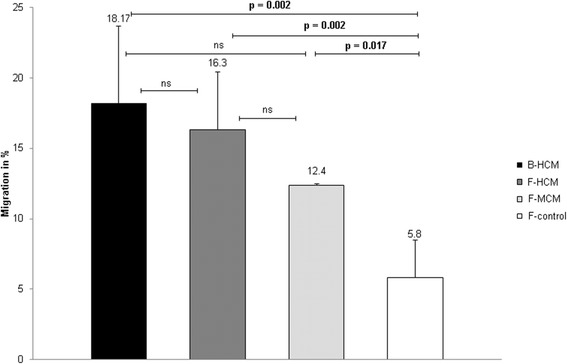
Migration (%) of human BMSC toward corresponding B-HCM and F-HCM, as well as F-MCM. Differences between migration of BMSC attracted by control (untreated) media or HCM and MCM were calculated using *t*-test. *p* < 0.05 statistically significant, ns not significant. Experiments were performed with different donors and passages four times (every time with three replicates per sample). B-HCM hypoxia conditioned media from BMSC, BMSC bone marrow stromal cells, F-control control media derived from skin fibroblasts, F-HCM hypoxia conditioned media from skin fibroblasts, F-MCM metabolically conditioned media from skin fibroblasts

We also analyzed F-HCM and F-MCM, generated from skin fibroblasts, with respect to BMSC migration toward this agent. F-HCM increased BMSC migration as efficiently as B-HCM (Fig. 2). Migration of BMSC was increased 2.1-fold (*p* = 0.017), which was less than migration induced by F-HCM (2.8-fold) but still significant in comparison to control media (F-control). In addition, we tested HCM from osteoarthritis fibroblasts which did not show increased chemoattractive ability (data not shown).

In summary we could clearly show that metabolically conditioned media derived from either human BMSC or skin fibroblasts have a similar chemoattractive capacity for BMSC as hypoxia conditioned media.

## Discussion

The main goal of tissue engineering is to offer a replacement for damaged tissues using implants, generated from cells, growth factors, and scaffolds. In our research we aim to attract bone-marrow-derived mesenchymal stroma cells (BMSC) into local bone defects in order to promote vascularization and healing with the help of hypoxia conditioned media (HCM) and metabolically conditioned media (MCM). Until now the key challenge in the clinical application of cell-based tissue engineering bone implants was the poor diffusion of oxygen into the tissue, making a functional blood vessel network a necessity [16]. Certain growth factors have been described to be crucial for bone fracture healing and are used for bone tissue engineering, most important of them bone morphogenic protein (BMP)-2 [17]. Angiogenic growth factors, such as vascular endothelial growth factor (VEGF), or placental growth factors are also crucial for fracture repair and are used to induce angiogenesis in bone tissue engineering constructs. Fibroblast growth factors and transforming growth factors act on osteogenesis and angiogenesis [18]. In bone development and repair, hypoxia signaling has a crucial role and many studies have focused on the link between angiogenesis and bone formation which is largely mediated by VEGF, the target gene of the HIF-1 pathway [19].

In our previous research we found that HCM contains a low concentration of VEGF (40–1990 pg/ml) and a high concentration of High mobility group box 1 (HMGB1) protein (8077–25,151 pg/ml) but has a higher chemoattractive capacity than recombinant VEGF (200 ng/ml) [15]. The key for effective migration of BMSC seems to be the combination of all factors present in HCM. In our studies we were able to further characterize and determine the angiogenic factors and cytokines that were present in the conditioned media. In our migration assays we were able to show for the first time a potent and cheap alternative to HCM that we termed MCM, which we were able to characterize by human cytokine array and ELISA for selected angiogenic factors. Proteins like Angiogenin, Keratinocyte Growth Factor (KGF), Pentraxin-3, Thrombospondin-1, Tissue Inhibitor of Metalloproteinases (TIMP-1), and urinary Plasminogen Activator (uPA) were quantified exactly and we were able to show that they are present in high concentrations in both HCM and MCM. Those factors play a crucial role in migration and vascularization.

At the same time our data suggest that the higher concentration of angiogenic factors in MCM does lead to a higher migration capacity (Fig. 1), as the chemoattraction of MCM is as efficient as that of HCM. The higher concentration of factors in MCM can be explained by the long incubation period of 2 weeks in the same media (starving) versus only 2 days of hypoxia which might lead to accumulation of the total concentration of each factor. In addition, different conditions (hypoxia and starving) might lead to different protein expression profiles. Furthermore, the cytokine arrays showed the presence of Stromal cell-derived factor-1 (SDF-1) in HCM and MCM. SDF-1 is crucial for homing and migration of multiple stem cell types associated with injury repair [20]. Its expression is promoted under hypoxic conditions [21]. SDF-1 also plays an essential role in directing BMSC migration to ischemic heart tissues [22].

Another interesting discovery was the absence of macrophage migration inhibitory factor (MIF) in B-HCM. MIF is usually overexpressed under various stress conditions such as ischemia, hypoxia, and oxidation, and it exerts an apparent cellular protective effect through repair of DNA damage, modulation of p53 gene expression, and reduction in oxidative stress [23, 24]. The reason for the absence of MIF might be that our BMSC were exposed to 1% hypoxia for 48 h instead of 3% for up to 24 h (conditions which are usually described in cell studies) [25], which might lead to a degradation of MIF. Interleukin-8 (IL-8) was also undetectable in B-MCM. However, it had been demonstrated that in human macrophages IL-8 protein secretion was rapidly increased within 2 h of hypoxic exposure [26]. IL-8 might also be degraded rapidly in the supernatants, which would explain its absence after 2 weeks of B-MCM incubation. The protein expression profile and the presence of certain proteins might also vary depending on the BMSC donor.

A further important finding was an obvious discrepancy between the amount of angiogenic factors present in B-conditioned and F-conditioned media and respective controls (Table 1). The number of factors in F-HCM and F-MCM was considerably higher than in B-conditioned media; at the same time, the migration assays shown in Fig. 2 clearly indicated that the high amount of factors in both F-conditioned media did not boost the migration capacity, as the B-HCM migration of 18.17% was still slightly higher than the F-HCM migration of 16.3% and the F-MCM migration of 12.4%. It can be assumed that a high number of factors might not be correlated directly with a higher chemoattractive effect of the conditioned media. The angiogenesis arrays also detected five additional factors which were upregulated in F-MCM and control and downregulated in F-HCM. This might be associated with a slightly reduced migration toward F-MCM in comparison to F-HCM. After characterization of our conditioned media we were able to conclude that all angiogenic factors which were present in B-MCM could be found in F-MCM, and while a lot of factors which were found in F-HCM were missing in B-HCM, however, this did not negatively affect its chemoattractive capacity.

Relative numbers of migrating cells were found to be slightly different depending on the source of the conditioned media. For the migration tests summarized in Fig. 1 (comparison of B-HCM and B-MCM) we used patient samples from early passages. For the experiments with conditioned media derived from fibroblasts we needed to repeat BMSC migration (as summarized in Fig. 2), because we wanted to make sure that these cells were all analyzed under exactly the same conditions. Taking the results from another experiment as internal control was of course not feasible. The relative numbers of migrating BMSC can vary depending on the donor, cell passage, and experimental setting. Here control media of different cell sources induced different levels of cell migration themselves without further conditioning. Control media derived from fibroblasts induced more migration than that derived from BMSC, perhaps due to the presence of more angiogenic factors per se (Table 1).

BMSC are known for their proangiogenic qualities and are currently being used to treat a wide variety of diseases in adults [27]. Stem cell therapy offers a great promise in the treatment of ischemic injuries [28], myocardial infarction [29], or hindlimb ischemia [30]. BMSC are also widely used in cell therapy because of efficient expansion, immune tolerance, and differentiation capacity [31]. However, often it is complicated to get access to human BMSC from patients. In order to make a step toward individualized therapy we decided to find out whether human BMSC would react to HCM and MCM produced by skin fibroblasts, as it is much more feasible to obtain skin fibroblasts from patients independent of age or physical condition. We determined that HCM and MCM produced by human skin fibroblasts are just as potent chemoattractive agents for human BMSCs as HCM and MCM derived from human BMSC. Both agents are able to provide highly significant increase of migration capacity and therefore can be used in the future to attract BMSC to the site of local injury.

## Conclusions

In this study we describe a cheap and efficient alternative to hypoxia conditioned media (HCM) termed metabolically conditioned media (MCM) which can be obtained from BMSC and skin fibroblasts. The chemoattractive capacity for BMSC is as efficient as that of HCM. MCM from BMSC and human fibroblasts contain high concentrations of angiogenic factors. Metabolic conditioning makes the access to chemoattractive agents easier, cheaper, and faster.

## Abbreviations

B-HCM: Hypoxia conditioned media from BMSC
B-MCM: Metabolically conditioned media from BMSC
BMSC: Bone marrow stromal cells
ELISA: Enzyme-linked immunosorbent assay
F-HCM: Hypoxia conditioned media from skin fibroblasts
F-MCM: Metabolically conditioned media from skin fibroblasts
HCM: Hypoxia conditioned media
IL-8: interleukin-8
MCM: Metabolically conditioned media
MIF: Macrophage migration inhibitory factor
SDF-1: Stromal cell-derived factor-1
